# Analytical Characterization and Pharmacokinetic Insight of Bioactive Compounds from *Champia parvula* and *Moringa oleifera* for Biocontrol of Blue Mold in Apple Fruits

**DOI:** 10.3390/plants14142104

**Published:** 2025-07-08

**Authors:** Eman A. Alwaleed, Mashail N. Alzain, Naglaa Loutfy, Amany A. El-Shahir

**Affiliations:** 1 Department of Botany and Microbiology, Faculty of Science, South Valley University, Qena 83523, Egypt; eme_biologist@sci.svu.edu.eg; 2 Department of Biology, College of Science, Princess Nourah bint Abdulrahman University, P.O. Box 84428, Riyadh 11761, Saudi Arabia; mnalzain@pnu.edu.sa

**Keywords:** gas chromatography, HPLC analysis, antifungal, phenolic compound, *Moringa oleifera*, *Penicillium*, blue mold, *Champia parvula*

## Abstract

The present study aimed to identify the active chemical compounds, mainly phenolic acids, of *Champia parvula* and *Moringa oleifera*, evaluate the pharmacokinetic properties of their primary compounds, and assess a novel method for the biocontrol of blue mold by evaluating the antifungal activity of both extracts. Gas chromatography (GC) and high-performance liquid chromatography (HPLC) were utilized to identify the active chemical compounds, mainly phenolic acids. GC illustrated the presence of long-chain aliphatic fatty acids like eicosanoic acid with the formation of oct-1-en-3-ol compounds attached. Catechin was the main bioactive component among the several bioactive compounds identified by HPLC analysis, exhibiting favorable pharmacokinetic behavior, including good absorption, distribution, and metabolic stability. According to the findings, both extracts had antifungal activity, but *C. parvula* extract (100 mg/mL) exhibited the strongest in vitro and in vivo antifungal activity, with the highest percentages of inhibition (disk diffusion method) against *Penicillium expansum*, *Penicillium crustosum*, and *Talaromyces atroroseus*, ranging between 62.67 and 100%. *C. parvula* extract (100 mg/mL) could fully inhibit the pathogenicity and aggressiveness of the five tested strains in apple fruits (in vivo). In conclusion, the extract from *C. parvula* and *M. oleifera* shows potential antifungal properties and a high phytochemical content.

## 1. Introduction

Apple (*Malus domestica* Borkh.) from the family Rosaceae is one of the most important fruit crops worldwide. It is high in antioxidants and fiber. Consuming apples is associated with a decreased risk of developing a variety of chronic illnesses, such as diabetes, heart disease, and cancer. Additionally, apples may help people lose weight and enhance their brain and gut health [1].

Apple is one of the most widely cultivated deciduous fruits in the world, apples are native to Central Asia and have since spread throughout the globe. China was the world’s top apple producer in 2024, followed by the US. In 2024, the world’s production of apples was 10.2 million tons. Egyptian apple production during 2024 was estimated to be about 934,414 tons according to Food and Agriculture Organization http://www.fao.org/faostat/en/#data/QC (accessed on 20 March 2025). 

Fungal infection is common in post-harvest fruits. It is caused by many necrotrophic fungi such as *Aspergillus* sp., *Alternaria* sp., *Botrytis cinerea*, *Fusarium* sp., *Penicillium* sp., and many other fungi. Compared to different biotic stresses, fungal infections are more common and have a more detrimental effect on agricultural productivity. Some of the worst famines in history have been related to fungal diseases [2,3,4].

Blue mold, which is caused by *Penicillium* spp., is one of the most common post-harvest decay diseases that affect pome fruit, such as apples, pears, and quinces, both in the short and long term. Microbes can easily infiltrate fruits due to their juicy nature [5]. When the fungus colonizes damaged fruits, it produces a soft, watery brown lesion with a distinct border, which is the first sign of blue mold degradation. In addition to rot, mycotoxins generated by these fungi are a significant cause of post-harvest loss [6,7].

Several strategies are needed for fighting fungal infections. Fungicides are the most popular technique. However, it is costly, pollutes the environment, and could eventually cause illnesses; furthermore, fungal strains become resistant [8]. As a result, efforts have been made to identify safe, environmentally friendly, and pathogen-specific alternatives to control infections. The potential of plant and algae extracts for the environmentally friendly management of fungal plant diseases was assessed through their use in vitro and in vivo experiments [4,9].

The cultivated species *Moringa oleifera* L., also known as the drumstick, is a member of the *Moringaceae* family [10]. Although it is currently widespread in many tropical and subtropical regions of the world, it originated in Pakistan, Afghanistan, and Egypt [11]. Moringa has been utilized in medicine as a nutritional supplement and in agriculture as a yield enhancer due to its remarkable nutritional and therapeutic properties [12]. Although much research has been carried out on the chemical components of *Moringa* and its potential medical uses, little is known about its antifungal properties. *Moringa* leaf extract (MLE) is a natural and sustainable source of plant growth-promoting compounds, growth regulators, osmoprotectants, antioxidants, secondary metabolites, antimicrobials, and mineral nutrients that increase plant resilience to stress [13,14,15].

Algal biomass offers numerous bioactive compounds that benefit the welfare of organisms globally [16,17]. Algae serve as a storehouse for various bioactive substances that can be used to create valuable products, ranging from life-saving medications to biomedical applications. Algae are used in biomedicine for antibacterial [18], antifungal [19], and antioxidant [20] uses. Globally, infectious diseases are a major contributor to high rates of morbidity and mortality. Seaweed has been used as a bioactive ingredient in nutraceutical applications, and several health products have been created from marine sources.

Phenolic compounds have been used to minimize the physiological harm that fungi cause to plants. According to [21], red seaweed, brown seaweed, and green seaweed (*Ulva* sp.) all contain phenolics like p-hydroxybenzoic acid and protocatechuic acid. *Moringa oleifera* also demonstrated rich phenolic acids and flavonoids. *M. oleifera* extract exhibits significant antioxidant activity both in vitro and in vivo [22]. *M. oleifera* leaves, especially, have the highest phenols and antioxidant activity compared with roots, barks, flowers, and seeds [23].

This study aimed to determine the phenolic profile, bioactive contents, and biosafety index of the *Moringa oleifera* and *Champia parvula* extracts, and to evaluate a novel method to control blue mold in post-harvested apple fruits by determining the antifungal activities of *C. parvula* and *M. oleifera* extracts against *Penicillium expansum* strain AP1 (PQ859555), *Penicillium crustosum* strain AP2 (PQ859337), *Talaromyces atroroseus* strain AP3 (PQ859549), *Penicillium expansum* strain AP4 (PQ859335), and *Penicillium expansum* strain AP5 (PQ859336). The effectiveness of the two extracts was evaluated in terms of (a) the use of gas chromatography (GC) and high-performance liquid chromatography (HPLC) to obtain insight into the active ingredients, (b) fungal growth inhibition (in vitro), and (c) apple fruit pathogenicity and aggressiveness (in vivo). As a result, these bioactive compounds could be used as a natural preservative or a component in future food products, serving as fungistatic or fungicidal agents.

## 2. Results

### 2.1. GC/MS Analysis of the Moringa oleifera and Champia parvula Water Extract

**Figure 1:**
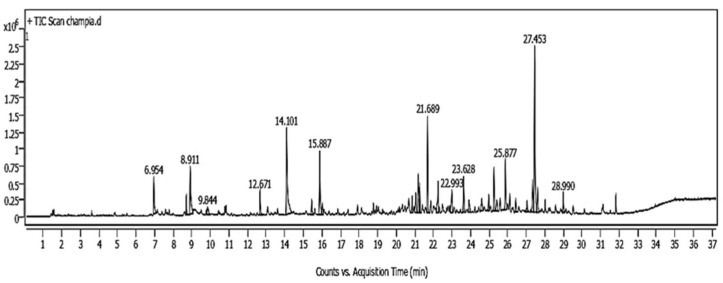
Compound recognized by GC-MS in *Champia parvula* water extract.

**Figure 2:**
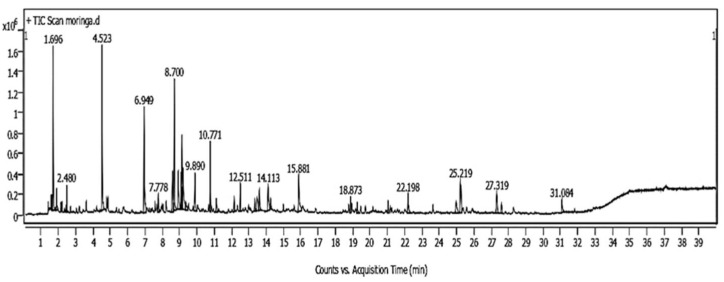
Compounds identified by GC-MS in *Moringa oleifera* water extract.

As shown in Figure 1 and Figure 2, the GC/MS chromatogram of the extracts of *Moringa oleifera* and *Champia parvula* showed 17 and 12 peaks, respectively. Table 1 displays the chemical composition of the *C. parvula* extract. The six most prevalent components were benzyl benzoate (RT = 27.45 min), heptadecane (RT = 25.88 min), isospathulenol (RT = 28.99 min), Anethole (RT = 15.88 min), β-hydroxyethyl phenyl ether (RT = 15.01 min) limonen-6-ol, pivalate (RT = 22.99 min), and benzyl alcohol (RT = 8.91 min). Compound name, molecular weight, peak area percentage, peak height percentage, and retention time were obtained from the library data and reported in Table 1 and Table 2. Correlating the presence of certain phenolics, fatty acids, and terpenes to the antifungal activity exhibited by seaweeds has been attempted. The triterpene squalene was the most predominant compound. However, as shown in Table 2 and Figure 2, seven chemicals were identified by gas chromatography–mass spectrometry based on their molecular formula and retention time in *M. oleifera* leaf extract. The most compounds identified included carbon disulfide, 2-ethylfuran, (E)-2-hexenal, benzaldehyde—psi.-cumene, 1,1′-oxydi-2-propanol, limonene, and 2,6-dimethyl-7-octen-2-ol.

### 2.2. Phenolic Compounds in Champia parvula and Moringa oleifera

**Figure 3:**
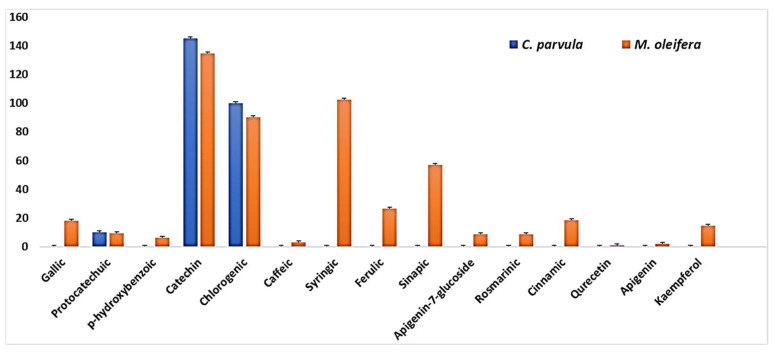
Quantification of phenolic acid from polar fractions of various accessions of *Champia parvula* and *Moringa oleifera* phenolic profile (μg/g).

In this study, two extracts, *C. parvula and M. oleifera*, were studied. Figure 3 shows the HPLC-DAD chromatograms of the *M. oleifera* and *C. parvula* phenolic compounds. Fifteen phenolic compounds were found and expressed as μg/g of *M. oleifera*, while three phenolic compounds were found in *C. parvula.* The chemical structures of the important compounds are shown in Table 3. The details of the peaks and phenolic compounds’ standards shown in the chromatograms are given in Supplementary Materials (Tables S1–S3, and Figure S1).

### 2.3. Pharmacokinetic Characteristics of Catechin by (ADMET)

**Figure 4:**
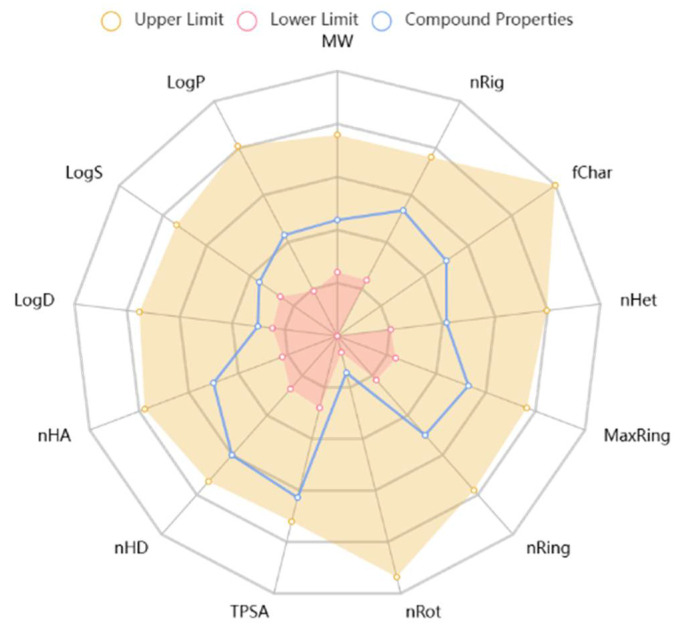
The radar graphic for the physiochemical features of catechin created by ADMET.

The study used the ADMET lab 2.0 tool to examine the pharmacokinetic properties of the predominant chemical in the extract of *Moringa oleifera* (catechin). Figure 4 displays the results as well as a radar image displaying the 13 different physicochemical properties. According to the findings, catechin possesses the physicochemical properties required for a pharmacological classification. Based on the data, the synthesized compounds’ physicochemical properties demonstrate that they adhered to the Lipinski rule of five (Table 3). This implies that they are appropriate for oral treatments due to their molecular structure.

A pharmacologically suitable molecule’s oral bioavailability increases when the Lipinski rule of five is followed. The molecular weight of catechin is 290.27 g/mol. This makes it better for oral management. Five hydrogen bond donors and six hydrogen bond acceptors (nHA) are present. Catechin has one rotatable bond. An equivalent drug candidate has a perfect charge range of −4 to 4. The topological polar surface area (TPSA) is 110.38 Å2. Figure 4 shows the oral bioavailability radar pattern predicted by Swiss ADME. This graphic shows a molecule’s flexibility, unsaturation, insolubility, lipophilicity, polarity, and size. Except for both compounds’ anomalous unsaturation, their physicochemical properties fit within the pink area. The chart’s pink area shows the ideal physicochemical environment for oral medicine.

The classification endpoints, the prediction probability values, are transformed into six symbols: 0–0.1 (---), 0.1–0.3 (--), 0.3–0.5 (-), 0.5–0.7 (+), 0.7–0.9 (++), and 0.9–1.0 (+++).

### 2.4. Solubility and Lipophilicity

Table 3 summarizes catechin’s water solubility and lipophilicity profiles. The solubility in water (Log S) is −2.14, which is higher than the range of −4.5 to 0.5 log mol/L. Log P is the log of the octanol/water partition coefficient, and it should be between 0 and 3. As a result of their partitioning into the lipid compartment, they display log P values of 0.83. A high gastrointestinal (GI) absorption rate has been demonstrated for catechin. It was determined that the bioavailability was 0.55. It was said to be able to pass through the blood–brain barrier. They are not P-glycoprotein substrates. Enzymes called cytochrome P450 have a role in catechin metabolism. It has been discovered that catechin inhibits CYP 1A2, CYP 2C19, and CYP 2C9.

### 2.5. In Vitro Antifungal Activity

**Figure 5:**
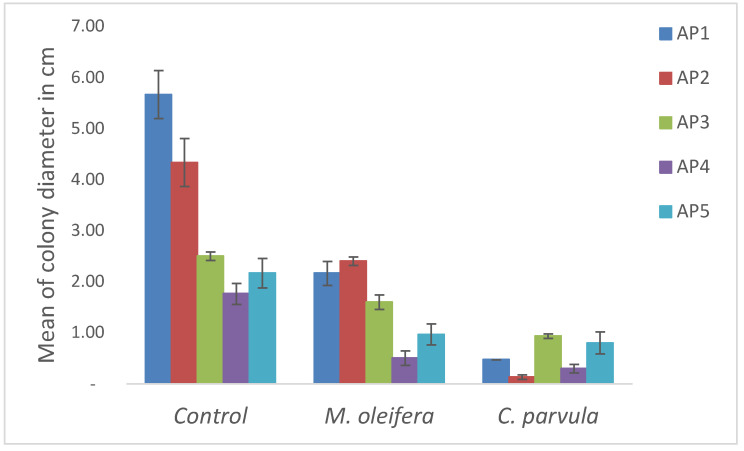
In vitro, antifungal activities of *Moringa oleifera* and *Champia parvula* extracts against *Penicillium expansum* strain AP1 (PQ859555), *Penicillium crustosum* strain AP2 (PQ859337), *Talaromyces atroroseus* strain AP3 (PQ859549), *Penicillium expansum* strain AP4 (PQ859335), and *Penicillium expansum* strain AP5 (PQ859336) after 10 days of incubation at 28 °C. Values are means of three replicates.

The antifungal activity of *M. oleifera* and *C. parvula* extracts against *P. expansum* strain AP1 (PQ859555), *P. crustosum* strain AP2 (PQ859337), *T. atroroseus* strain AP3 (PQ859549), *P. expansum* strain AP4 (PQ859335), and *P. expansum* strain AP5 (PQ859336) varied according to a growth inhibition assay technique. The mean colony diameter (cm) for growth inhibition was used to measure the antifungal activities after the ten days of incubation, and the percentages of inhibition were computed. The fungus without any extract treatment (control) had the largest colony diameter, as shown in Figure 5 and Table 4. This was increased by extending the incubation period; after 10 days, the range increased to between 1.77 and 5.67 cm. *C. parvula* extract had the smallest colony diameter, ranging from 0.13 to 0.9 cm, among all the tested fungal strains. Following a 10-day incubation period, the *M. oleifera* extract demonstrated notable antifungal efficacy against the five tested strains, with colony diameter (cm) values ranging from 0.5 to 2.4 cm. The colony diameters of AP1, AP2, AP3, AP4, and AP5 treated with *M. oleifera* extract were 2.17, 2.40, 1.60, 0.50, and 0.97 cm, respectively, following ten days of incubation. However, the extract from *C. parvula* exhibited strong antifungal activity against the five strains that were examined. The colony diameters after the 10-day incubation period for AP1, AP2, AP3, AP4, and AP5 treated with *C. parvula* extract were 0.47, 0.13, 0.93, 0.30, and 0.80 cm, respectively.

Following ten days of incubation, the inhibitory percentages of the *M. oleifera* and *C. parvula* extracts for the five strains subjected to testing were determined. The *C. parvula* extract generally exhibited the highest inhibition percentage values against the five tested isolates, ranging from 62.67% to 100%. The inhibition percentage reached 100% with the *P. expansum* strain AP1. Meanwhile, *M. oleifera* extract had inhibition percentage values ranging between 36 and 71.69% for the five tested isolates. *P. expansum* strain AP4 had the highest percentage of inhibition (71.69%) (Table 5). We could conclude that *C. parvula* extracts generally exhibited the strongest in vitro antifungal activity.

### 2.6. Fungal Pathogenicity and Aggressiveness on Apple Fruit (In Vivo Experiment)

Table 6 shows the effect of *M. oleifera* and *C. parvula* extracts on the pathogenicity and aggressiveness of *P. expansum* strain AP1 (PQ859555), *P. crustosum* strain AP2 (PQ859337), *T. atroroseus* strain AP3 (PQ859549), *P. expansum* strain AP4 (PQ859335), and *P. expansum* strain AP5 (PQ859336) strains on apple fruits. Both extracts decreased the proportion of infected fruits as compared to the controls. Both the positive and negative controls were 100% infected. The aggressiveness (lesions’ diameter) for negative control was 2.2 cm, while the aggressiveness for positive control for the five different tested strains ranged between 1.2 and 3.7 cm. The highest aggressiveness was detected in the positive control, specifically in AP5. Apple fruits treated with *M. oleifera* extract showed no infected fruits nor aggressiveness with AP1, AP2, AP3, and AP4 strains, but only with AP5; the pathogenicity was 20% with 0.3 cm aggressiveness. However, the apple fruits treated with *C. parvula* extract showed no pathogenicity or aggressiveness with the five tested strains. We can conclude that *C. parvula* extract was more effective in the in vivo experiment because it could fully inhibit the pathogenicity and aggressiveness of the five tested strains in apple fruits.

## 3. Discussion

Apples are particularly vulnerable to fungal contamination, which can happen at various times. Before harvest, it might impair the orchard tree, but after harvest, the most serious infections that cause fruit to deteriorate happen. Apple production has large economic losses due to fungal post-harvest infections, which are estimated to be between 30 and 40 percent in developing nations and as much as 60 percent in the worst situations. The possibility of mycotoxin accumulation in the fruit represents a significant risk of fungal infestation. Apple deterioration is caused by the mycotoxigenic organisms of genera such *Fusarium*, *Alternaria*, or *Penicillium* [48].

Several strategies are needed to manage blue mold disease. The most common technique is the use of fungicides. It is expensive, pollutes the environment, and may cause strain resistance [8]. As a result, attempts have been made to identify safe, environmentally friendly, and pathogen-specific alternative methods of controlling infections. The potential of plant and seaweed extracts for the environmentally friendly management of fungal plant diseases was assessed both in vitro and in vivo [4,9,49]. Numerous studies have demonstrated that various herbs and seaweeds include chemicals with antibacterial and antioxidant qualities that protect cells from oxidative stress and pathogens [50,51]. These antimicrobial agents function by destroying enzymes, preventing the synthesis of proteins, or altering the structure, function, or integrity of the cytoplasmic membrane [52].

The GC/MS chromatogram of *Moringa oleifera* and *Champia parvula* extracts showed 17 and 12 peaks, respectively. According to Parham et al. [50], seaweed has a high concentration of carotenoids. The most common component was the triterpene squalene. Aqueous plant extracts typically exhibit antimicrobial activity. Cavanagh et al. [46] observed that the availability of many chemicals that may interact antagonistically in their overall activities was the reason why aqueous extracts were different from other extracting solvents.

The dried *M. olifera* leaves and *C. parvula* had free phenolics, the majority of which were flavonols and derivatives (FVL). Within this fraction, the major groups were catechin and qurecetin; as illustrated by the earlier studies [53]. The chromatograms of both types showed catechin in high concentrations in *C. parvula* (145.14 μg/g) followed by *M. olifera* (134.6 μg/g). Catechin possesses a wide range of helpful properties such as antifungal activity, as well as antioxidant activity, which is beneficial in the treatment of cancer, neurological disorders, and cardiovascular diseases [54].

Similarly, to previous studies, our results revealed that *C. parvula* extract was shown to be the most effective type of in vitro antifungal activity, with the greatest inhibitory percentage values against *P. expansum* strain AP1 (PQ859555), *P. crustosum* strain AP2 (PQ859337), *T. atroroseus* strain AP3 (PQ859549), *P. expansum* strain AP4 (PQ859335), and *P. expansum* strain AP5 (PQ859336) strains, ranging between 62.67 and 100%. The crude extracts of numerous Chlorophyta, Phaeophyta, and Rhodophyta species include bioactive substances with antifungal action, such as proteins, carbohydrates, fatty acids (FAs), polyunsaturated fatty acids (PUFAs), antioxidants, amines, amides, and pigments, according to several studies [55,56,57,58,59,60].

Our findings indicate that *C. parvula* extract was more successful in the in vivo experiment since it completely inhibited the pathogenicity and aggressiveness of the five strains of apple fruits that were evaluated. No previous studies were found for the biocontrol of blue mold by *M. oleifera* and *C. parvula* in post-harvested apple fruits, so we can consider our study the first research on this topic. Nevertheless, there have been a few studies on the antifungal activity of some seaweed or medicinal plants with other fruit crops. According to El-Shahir et al. [61], methanolic extracts of *Ziziphus spina-christi* leaves and fruits showed strong antifungal activity against *Alternaria alternata*, *Alternaria citri*, and *Alternaria radicina* growth in tomato fruits, with varying percentages of inhibition at varying concentrations. For the three examined fungi, both extracts decreased the diameter of lesions (0–0.5 cm) and the percentage of infected fruits (0–30%). Fruit extract (C5 200 mg/mL) showed strong antifungal activity against *A. alternata*, *A. citri*, and *A. radicina* after 10 days of incubation, with colony diameters of 2.6, 3.5, and 3.5 cm, respectively. Also, the pathogenicity and aggressiveness (in vivo antifungal activity) of *Aspergillus niger*, *Botrytis cinerea*, and *Mucor irregularis* on strawberry fruits were investigated by [4] regarding extracts from *Sargassum cinereum* and *Padina boergesenii*. According to the aggressiveness assessment, all extracts were able to lower the proportion of infected strawberries and the size of the three fungal lesions (0.1–3 cm) on post-harvest strawberries compared to the control, with inhibition ranging between 10 and 100%. Additionally, they found an important positive correlation between *B. cinerea* and *M. irregularis*, which did not grow when treated with extracts of *S. cinereum* and *P. boergesenii* in an in vitro experiment. The two extracts, however, showed the least amount of growth inhibition in *A. niger*. According to research, a variety of plants and algae contain substances with antimicrobial and antioxidant properties that protect against infections and oxidative stress in cells [50,58].

## 4. Materials and Methods

### 4.1. Seaweed Collection

*Champia parvula* was collected at Abu Qir Bay on the Egyptian shore of Alexandria. Approximately 1.5 kg of fresh material was gathered and identified morphologically using standard taxonomic keys [62]. After sample collection, the samples were cleaned with fresh and distilled water to remove debris and related epiphytes. They were then dried for a week in the shade. The dried seaweed was ground into a powder for further extraction.

### 4.2. Plant Material

*Moringa oleifera* leaves were gathered from South Valley University in the Qena Governorate of Egypt. An electric blender was used to grind the leaves to a powder.

### 4.3. Preparation of Extracts

The powder of seaweed *Champia parvula* and *Moringa oleifera* leaves were boiled with water at 1:1 (*w*/*v*) for 2 h. The homogenized solution was filtered using Whatman filter paper No. 1. The supernatant obtained was a 100% algal and plant liquid extract. The liquid extracts were gathered in an airtight container and kept at 4 °C for further examination and use. For both extracts, a concentration of 100 mg/mL was utilized.

### 4.4. Gas Chromatography–Mass Spectrometry (GC-MS) Analysis

Gas chromatography (Agilent 8890 GC System, Agilent, Santa Clara, CA, USA), mass spectrometry (Agilent 5977B GC/MSD), and an HP-5MS fused silica capillary column (30, 0.25 mm film thickness) were used to examine *C. parvula* and *M. oleifera*. The oven was programmed to increase from 50 °C to 240 °C at a rate of 5 °C/min and then from 240 °C to 280 °C at a rate of 10 °C/min. It stayed at 280 °C for 10 min. Splitless GC helium moved 1.1 mL/min. 240 °C injection. The 70 eV electron impact mode (EI) mass spectra were obtained from 50 to 500 amu scans. The NIST 2020 (NIST 20) library matched mass spectra library data to identify individual peaks [16].

### 4.5. HPLC Analysis

Kim et al. [63] used an Agilent Technologies 1100 series liquid chromatograph (Agilent, Santa Clara, CA, USA) with an auto-sampler and diode-array detector for HPLC analysis. The Eclipse XDB-C18 analytical column (150 × 4.6 μm; 5 μm) features a C18 guard column. A and B were acetonitrile and 2% acetic acid in water (*v*/*v*). A gradient program of 100% B to 85% B in 30 min, 85% B to 50% B in 20 min, 50% B to 0% B in 5 min, and 0% B to 100% B in 5 min was run at 0.8 mL/min for 60 min. Peaks were observed at 280, 320, and 360 nm for benzoic acid derivatives, cinnamic acid derivatives, and flavonoids in a 50 μL injection volume. Before injection, all samples were filtered using a 0.45 μm Acrodisc syringe (Gelman Laboratory, Ann Arbor, MI, USA). Peaks with congruent retention durations and UV spectra were compared to standards.

### 4.6. Predicting Drug-Likeness Using Pharmacokinetics

The computational Biology & Drug Design Group’s open-source ADMET Lab 2.0 application (https://admetmesh.scbdd.com/, accessed on 8 June 2024) was used to determine catechin’s absorption, distribution, metabolism, elimination, and toxicity, according to [64].

### 4.7. Fungal Strains Collection and Identification

Investigations of antifungal activity were conducted using five strains: *Penicillium expansum* strain AP1 (PQ859555), *Penicillium crustosum* strain AP2 (PQ859337), *Talaromyces atroroseus* strain AP3 (PQ859549), *Penicillium expansum* strain AP4 (PQ859335), and *Penicillium expansum* strain AP5 (PQ859336). These fungi were isolated from contaminated apple fruits from various stores in Qena, Egypt. The strains that were tested were identified at South Valley University’s Faculty of Science, Botany, and Microbiology Department. The baiting method, as described by [3], was used to isolate fungus from rotten apple fruits. The fungi were initially identified by their micromorphological characteristics, growth texture, and colony patterns [65]. Their molecular identification validated the morphological identity of these fungi. NCBI-BLAST (https://blast.ncbi.nlm.nih.gov/Blast.cgi, accessed on 20 March 2025) analyzed the acquired rDNA sequences. The ITS rDNA sequences were searched using BLAST to confirm the morphological identification [66].

### 4.8. In Vitro Antifungal Activity (Disk Diffusion Method)

Fungal spores were grown on Petri dishes filled with potato dextrose agar (PDA) and incubated for seven days at 28 °C. In this experiment, one milliliter of each of the investigated extracts—*M. oleifera* and *C. parvula* were aseptically and separately introduced to a sterile melted PDA medium at a concentration of 100 mg/mL. Control plates (C), lacking extracts, were created. Each was carried out in triplicate. Once the plates had cooled, the fungal inoculums were placed on the agar surface.

All Petri dishes were incubated for ten days at 28 °C. Following this period, the fungal mycelium’s radial growth was measured. The average colony diameter (in centimeters) measured the antifungal activity. Each fungal strain’s inhibition percentage was determined after 10 days of incubation compared to mycelia growth in the control plate.

The percentage of inhibition was computed using the following formula:

Inhibition percentage = R − r/R × 100

where (r) denotes the radial growth of fungal mycelia on the plate treated with *Moringa oleifera* and *Champia parvula* extracts, and (R) denotes the radial growth of fungal mycelia on the control plate.

### 4.9. Fungal Pathogenicity and Aggressiveness on Apple Fruit (In Vivo Experiment)

The pathogenicity and aggressiveness of *P. expansum* strain AP1 (PQ859555), *P. crustosum* strain AP2 (PQ859337), *T. atroroseus* strain AP3 (PQ859549), *P. expansum* strain AP4 (PQ859335), and *P. expansum* strain AP5 (PQ859336) strains were studied in vivo to see if *M. oleifera* and *C. parvula* extracts had an inhibitory action according to [4] with modifications. We used uniformly sized and shaped, healthy apple fruits in the experiment. The fruits were rinsed under running water and then immersed in a 1% sodium hypochlorite solution for two minutes. Sterile distilled water was used to rinse and then dry them in a laminar flow hood. After applying 10 mL of extracts of *M. oleifera* and *C. parvula* at a concentration of 100 mg/mL to each 200 g fruit, the fruits were allowed to dry for 30 min in a laminar flow cabinet. A thin mist was created by holding the sprayer 30 cm from the fruit to achieve uniform distribution. Conidia of the tested fungi were removed from seven-day-old PDA culture media by flooding each Petri dish with 10 mL of sterile distilled water and scrubbing the surface with a glass rod. The suspension was increased to 100 mL (~1 × 10^7^ spores/mL) to be used for fungal spray inoculation. A 2 mL spore solution of AP1, AP2, AP3, AP4, and AP5 was used to inoculate each apple. Ten replicas were made for every treatment. Positive controls were fruits that were individually infected with each of the five strains. Fruits that were not infected were used as negative controls. The fruits were incubated for 10 days at 25 °C. Following the incubation period, the quantity of contaminated fruits was noted, and the size of the lesion was evaluated. The percentage of infected fruits was used to determine pathogenicity, and the size of the lesion was utilized to assess aggressiveness. The mean of two separate experiments was used to recode the results.

## Figures and Tables

**Table 1:** Compounds identified by GC-MS in *Champia parvula* water extract.

RT (min)	Compound	Mol. Weight	Peak Area (%)	Biological Activities
6.954	Benzaldehyde (C_6_H_5_CHO)	106.12	3.28	Antimicrobial, Antitumor, Anti-inflammatory, and Antioxidant Activity [24].
8.911	Benzyl alcohol (C_6_H_5_CH_2_OH)	108.14	5.63	Local Anesthetic Effect, and Antimicrobial and Antiparasitic Activity [25].
9.844	Ethyl octyl ether (C_10_H_22_O)	158.28	1.06	Use in Fragrance and Cosmetic Applications [26].
12.671	1-Nonanol (C_9_H_20_O)	144.25	2.09	Antimicrobial Activity and Insecticidal and Repellent Properties [27].
14.101	β-Hydroxyethyl phenyl ether (C_6_H_5_OC_2_H_4_OH)	138.16	12.19	Antimicrobial Activity and Antioxidant Effects [28].
15.887	Anethole (C_10_H_12_O)	148.20	6.12	Antimicrobial, Antioxidant, Anti-inflammatory, and Anticancer Activity [29].
21.689	Isospathulenol (C_15_H_24_O)	220.35	7.21	Anti-inflammatory Activity, Antimicrobial Properties and Cytotoxic and Anticancer Activity [30].
22.993	Limonen-6-ol, pivalate (C_15_H_24_O_2_)	236.35	2.46	Antimicrobial Activity and Potential Cytotoxic Effects [31].
23.628	Hexadecane (C_16_H_34_)	226.44	3.29	Limited Antimicrobial Effect [32].
25.877	Heptadecane (C_17_H_36_)	240.5	4.4	Antimicrobial Carrier Properties [33].
27.453	Benzyl Benzoate (C_14_H_12_O_2_)	212.24	16.71	Antimicrobial Activity and Scabicide and Pediculicide [34].
28.99	Hexahydrofarnesyl acetone (C_18_H_36_O)	268.5	1.69	Antimicrobial Activity and Potential Use in Aromatherapy and Fragrance [35].
RT (min): Retention time			Mol. Weight: Molecular weight	

**Table 2:** Compounds identified by GC-MS in *Moringa oleifera* water extract.

RT (min)	Phenolic Compound	Mol. Weight	Peak Area (%)	Biological Activities
1.696	Carbon disulfide (CS_2_)	76.15	6.84	Neurotoxicity [36].
2.480	2-Ethylfuran (C_6_H_8_o)	96.13	1.09	Hepatotoxicity and Neurotoxicity [37].
4.523	(E)-2-Hexenal (C_6_H_10_O)	98.14	9.09	Antimicrobial, Antifungal Activity, and Insecticidal Properties [38].
6.949	Benzaldehyde (C_6_H_5_CHO)	106.12	8.77	Antimicrobial, Antitumor, Anti-inflammatory, and Antioxidant Activity [24].
7.778	Psi.-Cumene (C_9_H_12_)	120.19	1.21	Neurotoxicity, Hepatotoxicity, and Nephrotoxicity [39].
8.602	1,1′-Oxydi-2-propanol (C_6_H_14_O_3_)	134.17	4.61	Anti-mosquito Activity [38].
8.700	Limonene (C_10_H_16_)	136.23	8.87	Antimicrobial, Antioxidant, and Anti-inflammatory Activity [40].
9.890	2,6-Dimethyl-7-octen-2-ol (C_10_H_20_O)	156.26	3.14	Anti-inflammatory Activity, Antimicrobial Properties, and Cytotoxic Activity [41].
10.771	Nonanal (C_9_H_18_O)	236.35	4.37	Antimicrobial Activity and Anti-inflammatory Effects [42].
12.51	1,1-Dimethoxy-2,2,5-trimethylhex-4-ene (C_11_H_22_O_2_)	186.29	1.82	Antimicrobial Activity and Antioxidant Potential [43].
14.113	β-Hydroxyethyl phenyl ether. (C_6_H_5_OC_2_H_4_OH)	138.16	3.23	Antimicrobial Carrier Properties [33].
15.881	Anethole (C_10_H_12_O)	148.20	4.25	Antimicrobial Activity [34].
18.873	Jasmone (C_11_H_16_O)	164.24	1.2	Antioxidant Compound [44].
22.198	Dihydroactinidiolide (C_11_H_16_O_2_)	180.24	1.98	Antimicrobial Activity, Antioxidant Activity, and Potential Neuroprotective Effects. [45].
27.319	Ambrox (C_16_H_28_O)	236.39	2.52	Antimicrobial Activity [46].
31.084	Palmitic acid (C_32_H_64_O_2_)	480.8	1.21	Metabolic Health, and Cardiovascular Disease [47].
RT (min): Retention time			Mol. Weight: Molecular weight	

**Table 3:** The physiochemical characteristics of catechin are predicated by ADMET.

Physicochemical Properties		Pharmacokinetics	
Formula	C15H14O6	GI absorption	---
Molecular weight	290.27 g/mol	BBB permeant	--
Num. heavy atoms	21	P-gp substrate	--
Num. arom. heavy atoms	12	CYP1A2 inhibitor	+
Fraction Csp3	0.20	CYP2D6 inhibitor	+++
Num. rotatable bonds	1	CYP3A4 inhibitor	--
Num. H-bond acceptors	6	Carcinogenicity (Three-class)	Non-required
Num. H-bond donors	5	AMES Toxicity	Non-AMES toxic
Molar Refractivity	74.33	Log *K*_p_ (skin permeation)	---
TPSA	110.38 Å^2^		
Water solubility		Lipophilicity	
Log *S* (ESOL)	−2.22	Log *P*_o/w_ (iLOGP)	1.36
Solubility	1.74 × 10 mg/mL; 5.98 × 10^−3^ mol/L	Log *P*_o/w_ (XLOGP3)	0.36
Class	Soluble	Log *P*_o/w_ (WLOGP)	1.22
Log *S* (Ali)	−2.24	Log *P*_o/w_ (MLOGP)	0.24
Solubility	1.66 × 10 mg/mL; 5.72 × 10^−3^ mol/L	Log *P*_o/w_ (SILICOS-IT)	0.98
Class	Soluble	Consensus Log *P*_o/w_	0.83
Log *S* (SILICOS-IT)	−2.14	Druglikeness	
Solubility	2.09 × 10 mg/mL; 7.19 × 10^−3^ mol/L	Lipinski	Yes
Medicinal Chemistry		Ghose	Yes
PAINS	1 alert: catechol_A	Veber	Yes
Brenk	1 alert: catechol	Egan	Yes
Leadlikeness	Yes	Muegge	Yes
Synthetic accessibility	3.50	Bioavailability Score	0.55

The prediction probability values are transformed into six symbols: 0–0.1 (---), 0.1–0.3 (--), 0.3–0.5 (-), 0.5–0.7 (+), 0.7–0.9 (++), and 0.9–1.0 (+++).

**Table 4:** In vitro, antifungal activities of *Moringa oleifera* and *Champia parvula* extract against *Penicillium expansum* strain AP1 (PQ859555), *Penicillium crustosum* strain AP2 (PQ859337), *Talaromyces atroroseus* strain AP3(PQ859549), *Penicillium expansum* strain AP4 (PQ859335), and *Penicillium expansum* strain AP5 (PQ859336) after 10 days of incubation at 28 °C. Plates of control (fungus only) are also shown.

Fungi	Control	*Moringa oleifera*	*Champia parvula*
AP1	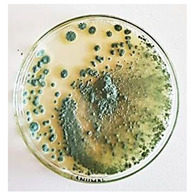	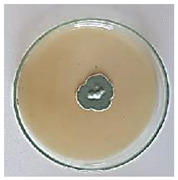	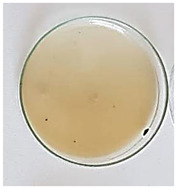
AP2	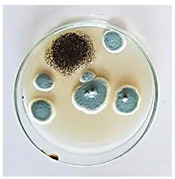	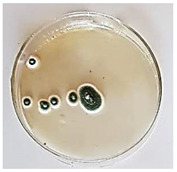	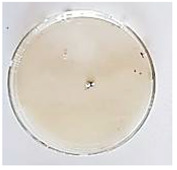
AP3	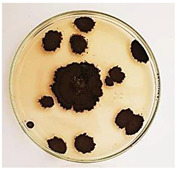	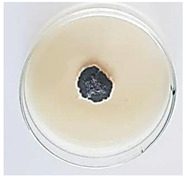	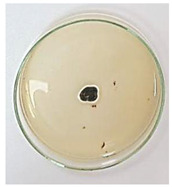
AP4	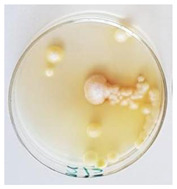	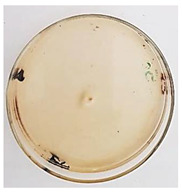	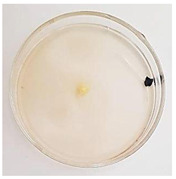
AP5	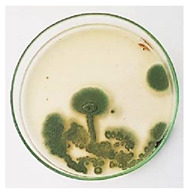	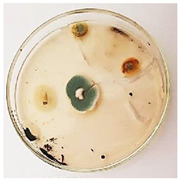	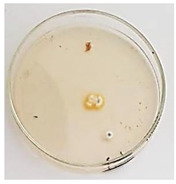

**Table 5:** Growth inhibition (%) of *Penicillium expansum* strain AP1 (PQ859555), *Penicillium crustosum* strain AP2 (PQ859337), *Talaromyces atroroseus* strain AP3 (PQ859549), *Penicillium expansum* strain AP4 (PQ859335), and *Penicillium expansum* strain AP5 (PQ859336) at 10 days of incubation with *Moringa oleifera* and *Champia parvula* extracts.

Fungi	*Moringa oleifera*	*Champia parvula*
AP1	61.73	100.00
AP2	44.57	96.93
AP3	36.00	62.67
AP4	71.69	83.01
AP5	57.37	64.70

Shades of green show high growth inhibition (%), shades of yellow show moderate growth inhibition (%), and shades of red show the low growth inhibition (%) of extracts.

**Table 6:** Effect of *Moringa oleifera* and *Champia parvula* extracts on pathogenicity and aggressiveness of *Penicillium expansum* strain AP1 (PQ859555), *Penicillium crustosum* strain AP2 (PQ859337), *Talaromyces atroroseus* strain AP3 (PQ859549), *Penicillium expansum* strain AP4 (PQ859335), and *Penicillium expansum* strain AP5 (PQ859336) on apple fruits.

Fungi/ Treatments	Extract	Pathogenicity % ^1^	Aggressiveness ^2^	Images
Negative control ^3^	No extract	100	2.2	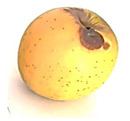
Positive control ^4^ AP1	No extract	100	1.2	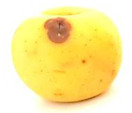
Positive control ^4^ AP2	No extract	100	1.3	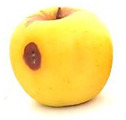
Positive control ^4^ AP3	No extract	100	1.5	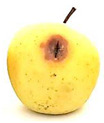
Positive control ^4^ AP4	No extract	100	2	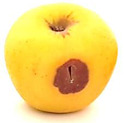
Positive control ^4^ AP5	No extract	100	3.7	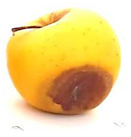
AP1	*M. oleifera*	0	0	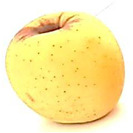
AP2	*M. oleifera*	0	0	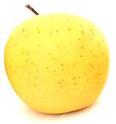
AP3	*M. oleifera*	0	0	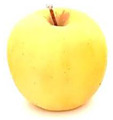
AP4	*M. oleifera*	0	0	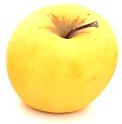
AP5	*M. oleifera*	20	0.3	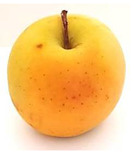
AP1	*C. parvula*	0	0	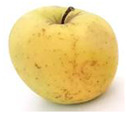
AP2	*C. parvula*	0	0	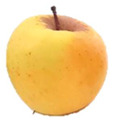
AP3	*C. parvula*	0	0	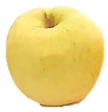
AP4	*C. parvula*	0	0	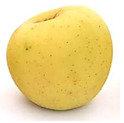
AP5	*C. parvula*	0	0	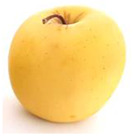

^(1)^ % of infected fruits. The mean of two independent experiments. ^(2)^ Mean lesion diameter (cm). Mean of ten replicates from two independent experiments. ^(3)^ Fruits without fungal inoculation or extracts. ^(4)^ Inoculated fruits untreated with *Champia parvula* and *Moringa oleifera* extracts.

## Data Availability

The data presented in this study is available on request from the corresponding author.

## Supplementary Materials

The following supporting information can be downloaded at: https://www.mdpi.com/article/10.3390/plants14142104/s1, Figure S1: Quantification of standard phenolic acid from polar fractions of various accessions of phenolic profile (μg/g); Table S1: Quantification of phenolic acid from polar fractions of multiple accessions of Champia parvula phenolic profile (μg/g); Table S2: Quantification of phenolic acid from polar fractions of various accessions of Moringa oleifera phenolic profile (μg/g); Table S3: Quantification of phenolic acid from polar fractions of various accessions of *Champia parvula* and *Moringa oleifera* phenolic profile (μg/g).
