# Supplementary material for: Analytical Characterization and Pharmacokinetic Insight of Bioactive Compounds from Champia parvula and Moringa oleifera for Biocontrol of Blue Mold in Apple Fruits
Source: Plants (Basel). 2025 Jul 8;14(14):2104. doi: 10.3390/plants14142104 (PMC12298388; doi:10.3390/plants14142104)

Supplementary Materials

Table S1: Quantification of phenolic acid from polar fractions of various accessions of *Champia parvula* Phenolic profile (µg/g)

Peak	RT	Area	Area Sum %	
1	6.954	1571313.57	3.28	Benzaldehyde
2	8.7	674898.24	1.41	Neodihydrocarveol
3	8.911	2697794.79	5.63	Benzyl alcohol
4	9.844	509480.23	1.06	Ethyl octyl ether
5	12.671	999946.88	2.09	1-Nonanol
6	14.101	5838686.05	12.19	β-Hydroxyethyl phenyl ether
7	15.452	622659.99	1.3	1-Decanol
8	15.887	2929121.12	6.12	Anethole
9	16.024	578605.17	1.21	Anethole
10	20.859	645580.37	1.35	Lanceol, cis
11	21.054	937504.02	1.96	2-Butyloxycarbonyloxy-1,1,10-trimethyl-6,9-epidioxydecalin
12	21.179	1998233.44	4.17	2,6,10-Trimethyltetradecane
13	21.265	1009576.74	2.11	Pentadecane
14	21.689	3453386.86	7.21	Isospathulenol
15	22.255	1560603.73	3.26	Crocetane
16	22.993	1175994.75	2.46	Limonen-6-ol, pivalate
17	23.628	1575393.13	3.29	Hexadecane
18	23.92	728660.53	1.52	Cedrol
19	24.607	734562.44	1.53	1-(2,6,6-Trimethyl-2-cyclohexen-1-yl)acetone
20	24.979	547694.19	1.14	Kharismal
21	25.265	1836564.67	3.83	Amberonne (isomer 2)
22	25.414	677285.61	1.41	2-Methylenecholestan-3-ol
23	25.877	2109402.02	4.4	Heptadecane
24	26.438	494076.22	1.03	1,1,7,7-Tetramethyl-s-hydrindacene
25	27.05	522074.29	1.09	2-Methyloctadecane
26	27.348	1480343.72	3.09	Ambrox
27	27.453	8002074.01	16.71	Benzyl Benzoate
28	27.611	1175706.04	2.45	Vertofix Coeur
29	28.99	809812.11	1.69	Hexahydrofarnesyl acetone

Table S2: Quantification of phenolic acid from polar fractions of various accessions of *Moringa oleifera* Phenolic profile (µg/g)

Peak	RT	Area	Area Sum %	
1	1.696	2308955.43	6.84	Carbon disulfide
2	1.902	318739.86	0.94	Methoxyacetic anhydride
3	2.48	366742.39	1.09	2-Ethylfuran
4	4.523	3067838.24	9.09	(E)-2-Hexenal
5	6.949	2959403.72	8.77	Benzaldehyde
6	7.778	408044.74	1.21	psi.-Cumene
7	8.602	1554424.03	4.61	1,1'-Oxydi-2-propanol
8	8.7	2994636.96	8.87	Limonene
9	8.923	1276050	3.78	Benzyl alcohol
10	9.083	804888.2	2.38	Diethylformal
11	9.14	1884714.88	5.58	Benzeneacetaldehyde
12	9.197	1603496.36	4.75	2-(2-Hydroxypropoxy)-1-propanol
13	9.358	365756.75	1.08	5-Hydroxy-4-hydroxymethyl-1-(1-hydroxy-1-isopropyl)cyclohex-3-ene
14	9.89	1060893.95	3.14	2,6-Dimethyl-7-octen-2-ol
15	10.771	1474121.22	4.37	Nonanal
16	11.114	469190.9	1.39	Phenylethyl Alcohol
17	12.156	325718.34	0.97	Decamethylcyclopentasiloxane
18	12.51	614464.16	1.82	1,1-Dimethoxy-2,2,5-trimethylhex-4-ene
19	13.346	312170.51	0.92	Ethyllinalool
20	13.478	724042.61	2.15	Ethyl maltol
21	13.615	596010.53	1.77	Decanal
22	14.113	1090044.86	3.23	β-Hydroxyethyl phenyl ether
23	14.256	344029.47	1.02	2-Oxaadamantane
24	15.881	1435952.88	4.25	Anethole
25	18.873	404097.1	1.2	Jasmone
26	19.263	327432.24	0.97	α-Cedrene
27	21.048	337066.89	1	trans-β-Ionone
28	22.198	667791.47	1.98	Dihydroactinidiolide
29	24.979	601182.53	1.78	Kharismal
30	25.219	1482823.49	4.39	Amberonne (isomer 2)
31	27.319	849894.41	2.52	Ambrox
32	27.594	311975.71	0.92	Vertofix Coeur
33	31.084	408847.17	1.21	Palmitic acid

Table S3: Quantification of phenolic acid from polar fractions of various accessions of *Champia parvula* and *Moringa oleifera* Phenolic profile (µg/g)

**Phenolic Compound**	*Champia parvula*	*Moringa olifera*
Gallic	ND	17.98
Protocatechuic	8.48	9.36
Gentisic	ND	ND
p-hydroxybenzoic	ND	6.30
Cateachin	424.62	346.84
Chlorogenic	ND	118.32
Caffeic	ND	5.00
Syringic	118.44	152.57
Vanillic	ND	ND
Ferulic	ND	26.62
Sinapic	ND	56.49
Rutin	ND	ND
p-coumaric	ND	ND
Apigenin-7-glucoside	ND	7.75
Rosmarinic	ND	7.82
Cinnamic	ND	17.64
Qurecetin	202.22	210.57
Apigenin	ND	2.51
Kaempferol	ND	14.28
Chrysin	ND	ND

Figure S1: Quantification of standard phenolic acid from polar fractions of various accessions of Phenolic profile (μg/g)

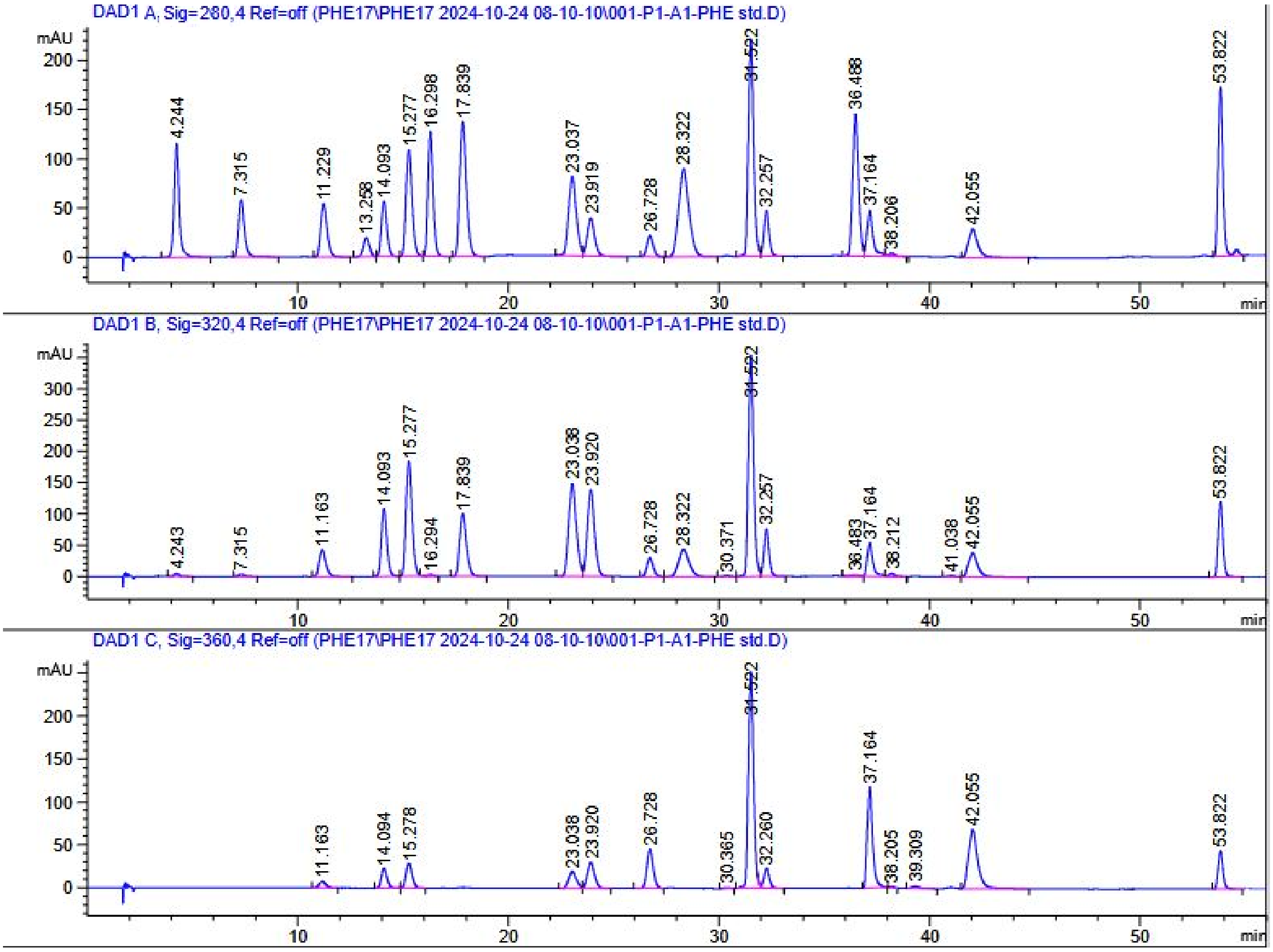

Supplement: Supplementary file 1 [file plants-14-02104-s001.zip › plants-3677518-supplementary-RESUBMIT-fig S1-revised.pdf]
